# The Plasmodium berghei serine protease PbSUB1 plays an important role in male gamete egress

**DOI:** 10.1111/cmi.13028

**Published:** 2019-04-29

**Authors:** Tomasino Pace, Felicia Grasso, Grazia Camarda, Catherine Suarez, Michael J. Blackman, Marta Ponzi, Anna Olivieri

**Affiliations:** ^1^ Dipartimento di Malattie Infettive Istituto Superiore di Sanità Rome Italy; ^2^ Malaria Biochemistry Laboratory The Francis Crick Institute London UK; ^3^ Faculty of Infectious and Tropical Diseases London School of Hygiene and Tropical Medicine London UK

**Keywords:** egress, gametocytes, malaria, Plasmodium berghei, SERA3, SUB1

## Abstract

The *Plasmodium* subtilisin‐like serine protease SUB1 is expressed in hepatic and both asexual and sexual blood parasite stages. SUB1 is required for egress of invasive forms of the parasite from both erythrocytes and hepatocytes, but its subcellular localisation, function, and potential substrates in the sexual stages are unknown. Here, we have characterised the expression profile and subcellular localisation of SUB1 in *Plasmodium berghei* sexual stages. We show that the protease is selectively expressed in mature male gametocytes and localises to secretory organelles known to be involved in gamete egress, called male osmiophilic bodies. We have investigated PbSUB1 function in the sexual stages by generating *P*. *berghei* transgenic lines deficient in PbSUB1 expression or enzyme activity in gametocytes. Our results demonstrate that PbSUB1 plays a role in male gamete egress. We also show for the first time that the PbSUB1 substrate PbSERA3 is expressed in gametocytes and processed by PbSUB1 upon gametocyte activation. Taken together, our results strongly suggest that PbSUB1 is not only a promising drug target for asexual stages but could also be an attractive malaria transmission‐blocking target.

## 1 INTRODUCTION

Malaria is a devastating disease with 216 million cases globally and 445,000 lethal outcomes in 2016 (Organization WH, 2017), mainly in children under the age of 5. The disease is caused by intracellular parasites of the *Plasmodium* genus, transmitted by the bite of anopheline mosquitoes. After an initial reproductive cycle in the liver, malaria parasites invade erythrocytes, where they either multiply asexually by forming schizonts or differentiate into gamete precursors called gametocytes. Asexual blood stages are responsible for the clinical manifestations of the disease, whereas gametocytes are essential for parasite transmission. When mature gametocytes are ingested by mosquitoes during a blood meal, they differentiate into gametes and egress from the host red blood cell by an inside‐out mode of egress (Sologub et al., 2011). Each female gametocyte forms a single macrogamete, whereas male gametocytes undergo a drastic transformation, known as exflagellation, by forming eight flagellar microgametes. Male gametes contact surrounding erythrocytes, forming so‐called “exflagellation centres,” in which fertilisation of the female takes place. Gamete egress follows a fixed programme, requiring discharge of secretory organelles called osmiophilic bodies (OBs) followed by inside‐out disruption of the parasitophorous vacuole membrane (PVM) and erythrocyte membrane (Ponzi et al., 2009; Sologub et al., 2011; Talman et al., 2011). This entire process is crucial for fertilisation and zygote formation and represents a major bottleneck in the *Plasmodium* life cycle, involving an approximate 300‐fold loss of parasite abundance (Kuehn & Pradel, 2010).

In male *Plasmodium berghei* gametocytes, OBs are club‐shaped, electron‐dense vesicles that are smaller than the oval‐shaped female OBs (Olivieri et al., 2015). Because of their gender‐specific morphological features, these vesicles are named male osmiophilic bodies (MOBs; Olivieri et al., 2015). Upon gametocyte activation, OBs accumulate at the gametocyte plasma membrane in multiple foci prior to secreting their contents into the parasitophorous vacuole (PV). In contrast, MOBs cluster together to form larger structures and then release their contents at only a few focal points (Olivieri et al., 2015). These different behaviours likely reflect specific mechanisms of vesicle discharge. A specific MOB marker was recently described in Plasmodium yoelii (Tachibana, Ishino, Takashima, Tsuboi, & Torii, 2018).

The threat of emerging malaria strains resistant to currently available drugs has made the search for novel drug targets compelling. In particular, the scientific community has recently acknowledged an urgent need for transmission‐blocking drugs targeting the sexual stages. Drugs effective against gametocytes/gametes, used in combination with drugs against asexual stages, would have the dual advantage of lowering parasite transmission and preventing the spread of drug‐resistant parasites. The subtilisin‐like protease SUB1 is a *Plasmodium* serine protease that is expressed both in the liver and blood schizonts and then secreted into the PV from vesicular organelles called exonemes just prior to schizont rupture (Yeoh et al., 2007). The proteolytic activity of SUB1 has been shown to be important for maturation and egress of invasive merozoites from both infected erythrocytes (Thomas et al., 2018; Yeoh et al., 2007) and hepatocytes (Tawk et al., 2013). During Plasmodium falciparum merozoite egress, PfSUB1 carries out the maturation of a family of papain‐like proteins called the serine repeat antigens (SERAs), whereas for merozoite maturation, the role of SUB1 is to undertake the processing of several merozoite surface proteins (Yeoh et al., 2007). Orthologs of *sub1* are found in every *Plasmodium* species examined, suggesting a conserved functional role.

In P. falciparum, maturation of PfSUB1 has been described in detail. The protease is synthesised as a preprotein that undergoes maturation by proteolytic processing in two consecutive steps: first, in an autocatalytic conversion, to a 54‐kDa form called p54, by the release of a prodomain (Sajid, Withers‐Martinez, & Blackman, 2000), and finally, by means of the P. falciparum protease plasmepsin X, to a terminal 47‐kDa species called p47 (Nasamu et al., 2017; Pino et al., 2017). In *P*. *berghei*, a similar processing pattern has been shown, with a ~70‐kDa fragment likely corresponding to full‐length PbSUB1 and a doublet at 44–47 kDa likely corresponding to the two processed forms of PbSUB1 (Suarez, Volkmann, Gomes, Billker, & Blackman, 2013). The subtilisin prodomain functions as an intramolecular chaperone, being essential for correct folding of the catalytic domain and for enzyme maturation (Jean, Hackett, Martin, & Blackman, 2003). Like many subtilisin prodomains, the SUB1 prodomain is a potent and highly selective inhibitor of SUB1 protease activity: it possesses inhibition constants in the nanomolar range and does not inhibit other subtilisin‐like serine proteases (Jean et al., 2003).

It was recently shown that PbSUB1 is also expressed in *P*. *berghei* sexual stages (Pino et al., 2017), but the function of the enzyme in gametocytes/gametes and its potential substrates in these stages are still unknown. Here, we show that PbSUB1 is selectively expressed in male *P*. *berghei* gametocytes and that it localises to MOBs (Olivieri et al., 2015). Using conditional depletion of PbSUB1 expression or enzyme activity, we show that the protease plays a role in male gamete egress. Our results strongly suggest that PbSUB1 is not only a promising drug target for asexual stages but could also be an attractive malaria transmission‐blocking target.

## 2 RESULTS

### 2.1 PbSUB1 is expressed in both asexual and sexual stage parasites and localises to MOBs in P. berghei gametocytes

**Figure 1:**
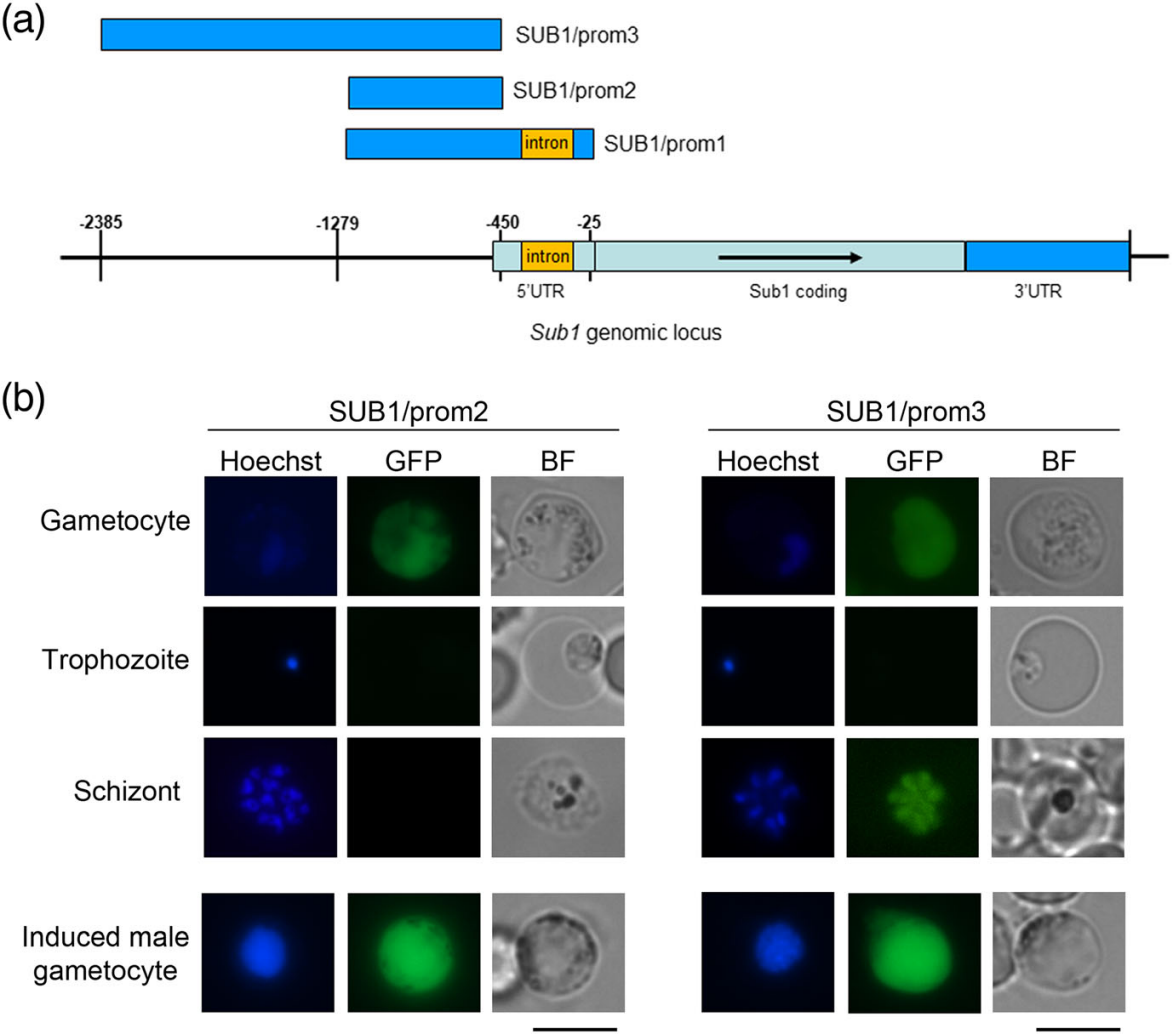
Sub1 promoter analysis. (a) Schematic illustrating sub1 genomic locus; in blue are highlighted the regions cloned in the SUB1/prom plasmids. (b) In vivo imaging of SUB1/prom gametocytes from synchronous infections and SUB1/prom schizonts from in vitro culture. The SUB1/prom2 line is shown as an example of the gametocyte‐specific GFP expression in both SUB1/prom1 and SUB1/prom2 lines. In the SUB1/prom3 line, GFP signal was detected also in mature schizonts. In all SUB1/prom lines, at 10 min postinduction, GFP fluorescence intensity increased in activated male gametocytes, with clearly identifiable replicating nuclei. All the images were acquired with the same settings. Nuclei are stained with Hoechst. BF, bright field; GFP, green fluorescent protein. Scale bar 5 μm

To investigate the regulation of *sub1* gene expression in gametocytes, we generated *P*. *berghei* transgenic lines in which DNA sequences upstream of the *pbsub1* start codon were cloned upstream of a reporter gene (green fluorescent protein [GFP]) and integrated into a silent locus (Figure S1), previously successfully used to integrate constructs into the *P*. *berghei* genome (Kooij, Rauch, & Matuschewski, 2012). The first two constructs, named SUB1/prom1 and SUB1/prom2, included the DNA sequence spanning the region between 1,279 bp and either 25 or 450 bp upstream of the *pbsub1* ATG, respectively, including or excluding an intron present in the *pbsub1* 5' UTR (Figure 1a). The resulting transgenic parasite lines were both found to specifically display reporter gene expression in the cytoplasm of gametocytes, with no detectable expression in asexual blood stage parasites (SUB1/prom2 expression is shown in Figure 1b, as an example). We then generated a third construct, where a larger DNA segment, extending from 2,385 to 450 bp upstream of the *pbsub1* ATG was fused to *gfp*. In the obtained transgenic line, named SUB1/prom3, the reporter gene was expressed both in gametocytes and schizonts (Figure 1b). These results suggested that the *pbsub1* gene includes two distinct regulatory regions: one, closest to the ATG, that specifically drives gene expression in the sexual stages and another, further upstream, necessary to drive expression also in asexual stages. Interestingly, in all the SUB1/prom lines, the GFP fluorescence signal significantly increased in activated male gametocytes (Figure 1b), indicating up‐regulation of *pbsub1* gene expression during male gamete formation.

**Figure 2:**
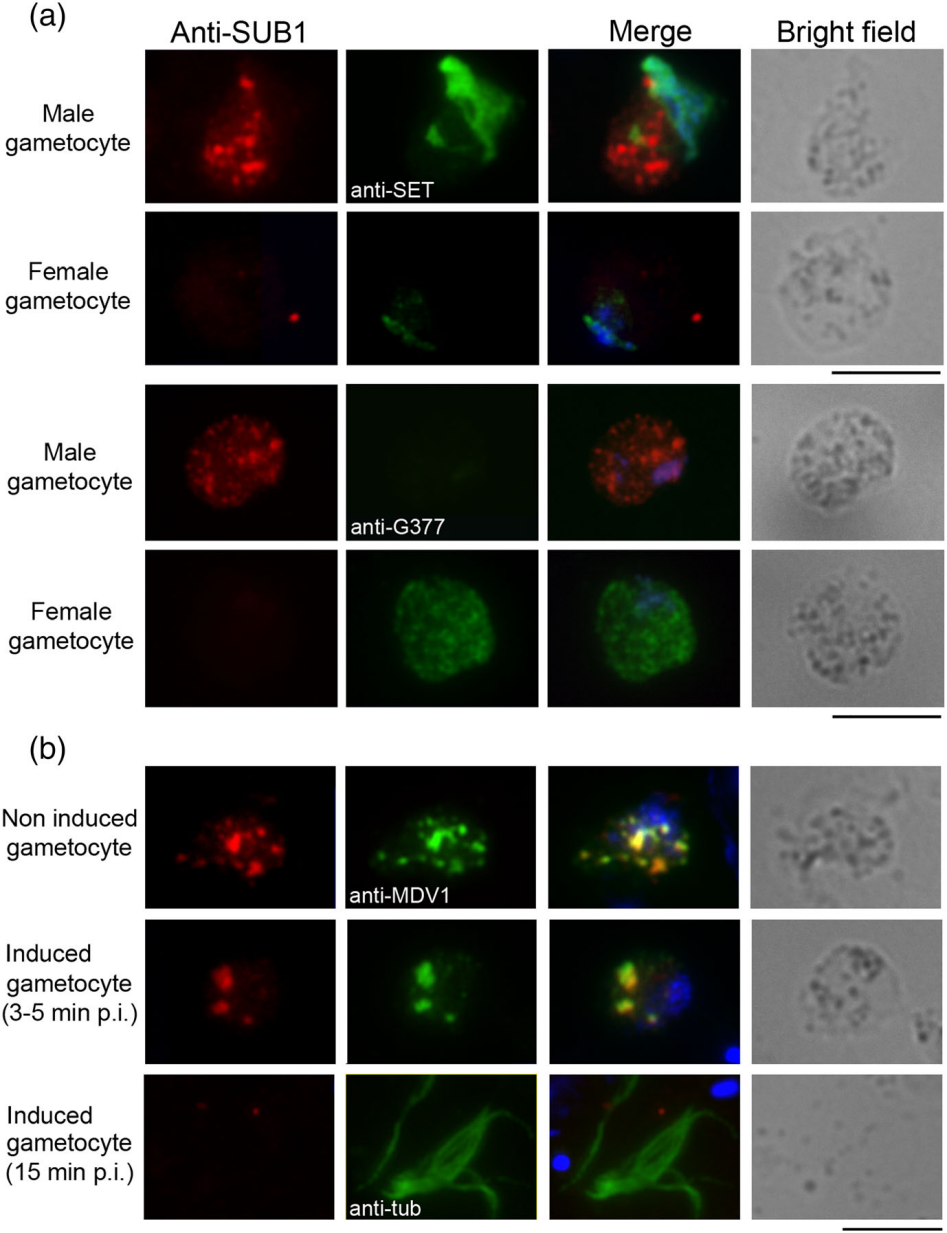
PbSUB1 is specifically expressed in male gametocytes and localises to MOBs. IFA of purified Plasmodium berghei gametocytes with anti‐SUB1 antibody. (a) Anti‐SET antibody was used as a male gametocyte marker and anti‐G377 antibody as a female gametocyte marker. The anti‐SUB1 fluorescence signal is present in gametocytes positive for the anti‐SET signal and negative for the anti‐G377 signal. (b) Anti‐MDV1 is an OB marker and anti‐alpha‐tubulin antibody stains male gamete flagella; nuclei are stained with DAPI; 3–5 min after induction to form gametes, MOBs tend to coalesce. By 15 min postinduction, when male gametes are released, the PbSUB1 signal is no longer detectable. Scale bar 5 μm. IFA, immunofluorescence assay; G377, Plasmodium gametocyte protein 377; MDV1, male development protein 1; p.i., postinduction; SET, SE translocation proto‐oncogene‐like protein

We next investigated the subcellular localisation of PbSUB1 in mature *P*. *berghei* gametocytes by immunofluorescence (IFA) using an anti‐PbSUB1 antibody (Suarez et al., 2013). Antibody specificity was confirmed in gametocytes by double IFA with an anti‐HA monoclonal antibody on a *P*. *berghei* line in which the endogenous *pbsub1* gene had been modified with a triple HA‐tag at the C‐terminal end (Suarez et al., 2013). The two signals colocalised in gametocytes, producing a punctate pattern (Figure S2). Double IFA with antibodies to the nuclear protein SE translocation proto‐oncogene like, which is highly abundant in male gametocytes (Pace et al., 2006) and with the female gametocyte marker G377 (Olivieri et al., 2015), showed that PbSUB1 expression in gametocytes is male specific (Figure 2a). Of 100 SUB1‐positive gametocytes examined, none showed a fluorescence signal with the G377 antibody. Colocalisation experiments with an antibody against the male development protein 1 (MDV1), a gametocyte‐specific protein associated with OBs in both *P*. *berghei* male and female gametocytes (Ponzi et al., 2009), showed that PbSUB1 specifically localises to these secretory organelles in males (MOBs; Figure 2b) with a Pearson's correlation coefficient of .94 (*P* < .001). Further examination showed that PbSUB1 follows the typical MOB pattern, being released upon gametocyte activation and not detectable in mature male gametes (Figure 2b).

### 2.2 PbSUB1 enzyme activity is required for male gamete egress

**Figure 3:**
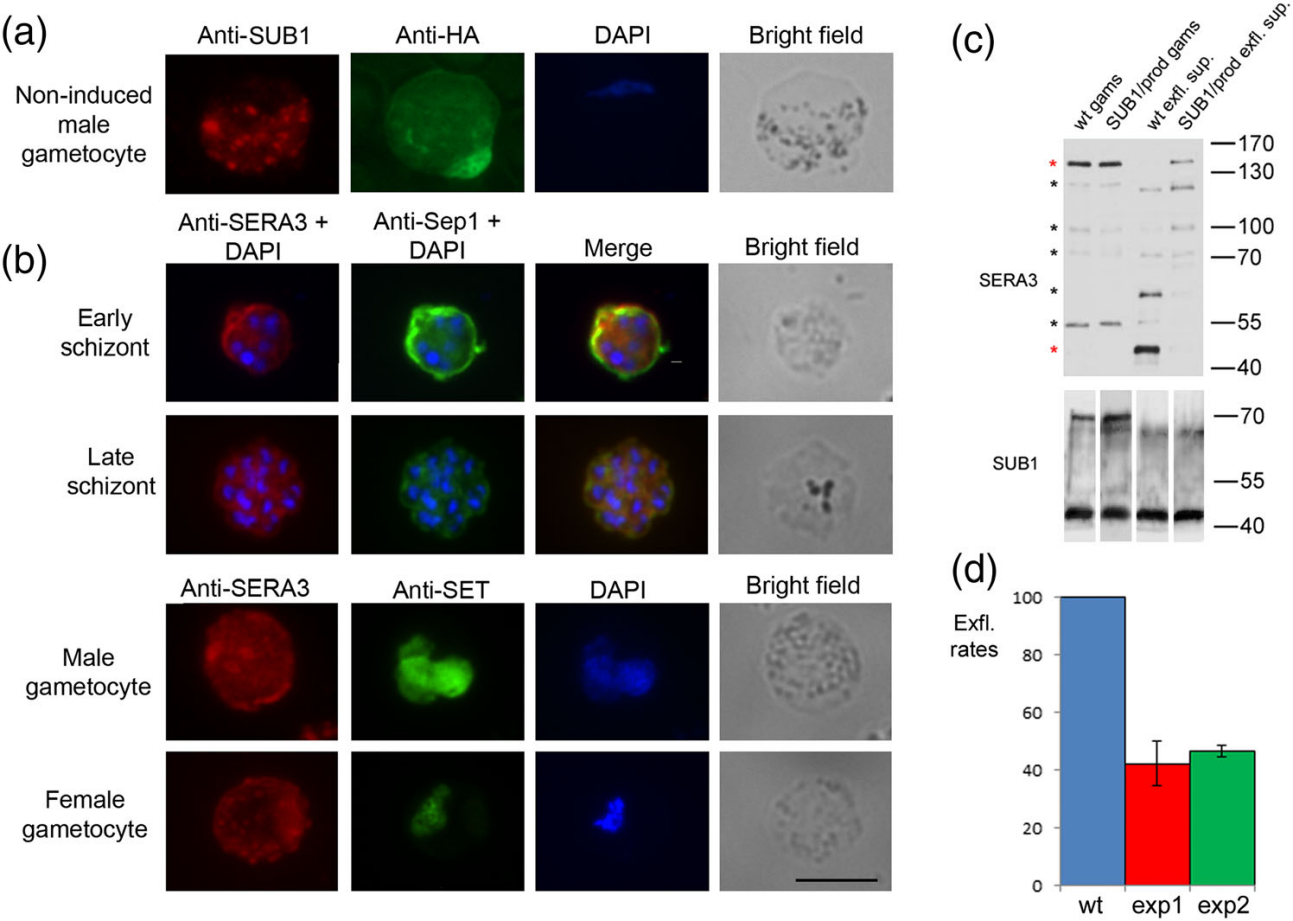
Transgenic expression of the PbSUB1 prodomain reduces endogenous PbSUB1 protease activity and inhibits exflagellation. (a) IFA of the transgenic line SUB1/prod with an anti‐HA antibody. Anti‐PbSUB1 was used as an OB marker. Nuclei are stained with DAPI. Scale bar 5 μm. (b) IFA of Plasmodium berghei blood stages with anti‐SERA3. Anti‐Sep1 antibody was used as a PVM marker; anti‐SET antibody was used as a gender marker; nuclei are stained with DAPI. Scale bar 5 μm. (c) Western blot analysis with anti‐PbSERA3 of extracts from 5 × 10^6^ wt gametocytes (gams) and 5 × 10^6^ SUB1/prod gametocytes, wt exflagellation supernatants (exfl. sup.) from 5 × 10^6^ gametocytes, and SUB1/prod exflagellation supernatants from 5 × 10^6^ gametocytes. Anti‐SUB1 was used as a loading control. Asterisks highlight the seven main processing products: PbSERA3‐130, PbSERA3‐110, PbSERA3‐100, PbSERA3‐72, PbSERA3‐55, PbSERA3‐65, and PbSERA3‐48. Red asterisks highlight the full‐length protein and the terminal processing product PbSERA3‐48. This experiment was conducted in two biological replicates, both showing the same processing pattern in gametocytes and exflagellation supernatants. (d) Exflagellation rates of the SUB1/prod line in two independent experiments, each performed in triplicate, at 15 min postinduction, expressed as a percentage of exflagellation rates in the wt line (Student's t test: p_exp1_ < 0.005 and p_exp2_ < 1.02 × 10^−5^; error bars represent +/− standard deviation of the mean value)

To investigate whether the proteolytic activity of PbSUB1 is essential for gamete egress, we took advantage of the capacity of the SUB1 prodomain to inhibit with high specificity the proteolytic activity of its cognate catalytic domain. To do this, we produced a transgenic *P*. *berghei* line, named SUB1/prod, expressing an HA‐tagged extra copy of the prodomain fused to the 5′ flanking region and the first 90 residues of the *mdv1* gene, in order to express the chimera in gametocytes and target the prodomain to OBs (Figure S3a). After confirming the integration event in clones of the SUB1/prod line (Figure S3b), the expression profile of the HA‐tagged PbSUB1 prodomain chimera was analysed by IFA and western blot (Figure S4). This showed that the transgene was specifically expressed in gametocytes, but the HA‐tagged prodomain did not colocalise with PbSUB1, suggesting that additional signals are required to traffic the chimera to MOBs (Figure 3). In male gametocytes, the transgenic prodomain showed a peripheral distribution with thickening of the signal in some areas around the parasite (Figure S4).

To assess whether the endogenous PbSUB1 proteolytic activity was inhibited as anticipated in the SUB1/prod line, we preliminarily investigated whether proteolytic processing of the PV protein PbSERA3, known to be a substrate of PbSUB1 during egress of merozoites from *P*. *berghei* liver schizonts (Tawk et al., 2013), also occurred in the sexual stages. The expression profile and subcellular localisation of PbSERA3 has not previously been investigated in *P*. *berghei* blood stages, although its orthologue in P. falciparum, PfSERA6, undergoes maturation by PfSUB1 in asexual blood stages (Ruecker et al., 2012; Thomas et al., 2018). As shown in Figure 3b, SERA3 is expressed in *P*. *berghei* blood schizonts, showing a peripheral localisation. It was also found to be expressed in gametocytes, displaying a diffuse signal, stronger in the cell periphery in both sexes; in addition, female gametocytes also showed a punctate pattern in the cytoplasm.

To investigate proteolytic processing of PbSERA3 by PbSUB1, noninduced gametocytes and supernatants from purified activated gametocytes were collected from the transgenic SUB1/prod and its parental parasite line and analysed by western blot (Figure 3c). Nonactivated gametocytes from both the wt and transgenic lines showed the same processing pattern, consisting of the PbSERA3‐130 precursor and the intermediate maturation products PbSERA3‐110, PbSERA3‐100, PbSERA3‐72, and PbSERA3‐55 (Ruecker et al., 2012; Schmidt‐Christensen, Sturm, Horstmann, & Heussler, 2008). In supernatants of exflagellating wt gametocytes, two further processing products were visible, at around 63 and 48 kDa, respectively, the latter being the final maturation product (Ruecker et al., 2012). The 63‐kDa band, not previously described, could be a processing product specific to sexual stages. The 55‐kDa band, unexpectedly disappearing from the SUB1/prod exflagellation supernatants, could remain partially associated to PVM, as previously reported for the SERA3 orthologue in P. falciparum, PfSERA6 (Ruecker et al., 2012). Both the 63‐ and 48‐kDa bands were prominent in supernatants from the wt line but were barely detectable in those of the activated SUB1/prod line; in contrast, the full‐length protein was undetectable in supernatants from the wt parasites, whereas it was conspicuous in supernatants of the SUB1/prod line. This suggested that (a) the two terminal PbSERA3 proteolytic processing events occur during gamete egress; (b) these processing events are PbSUB1 dependent in *P*. *berghei* sexual stages; and (c) PbSERA3 processing is significantly reduced in SUB1/prod gametocytes, demonstrating that in this line, PbSUB1 activity is strongly impaired. Although PbSUB1 is only expressed in male gametocytes, its substrate PbSERA3 is expressed in both genders. There may be several explanations for this, including the fact that expression of the protein in female gametocytes may be redundant or that its function in females does not require proteolytic maturation. Having confirmed inhibition of endogenous PbSUB1 activity in the SUB1/prod line, the efficiency of male gamete formation was evaluated in this line by counting exflagellation centres in vivo. As shown in Figure 3d, male gametes of the SUB1/prod line showed a significant reduction in exflagellation rates, compared with wt gametes, indicating that PbSUB1 enzymatic activity plays an important role in male gamete formation.

**Figure 4:**
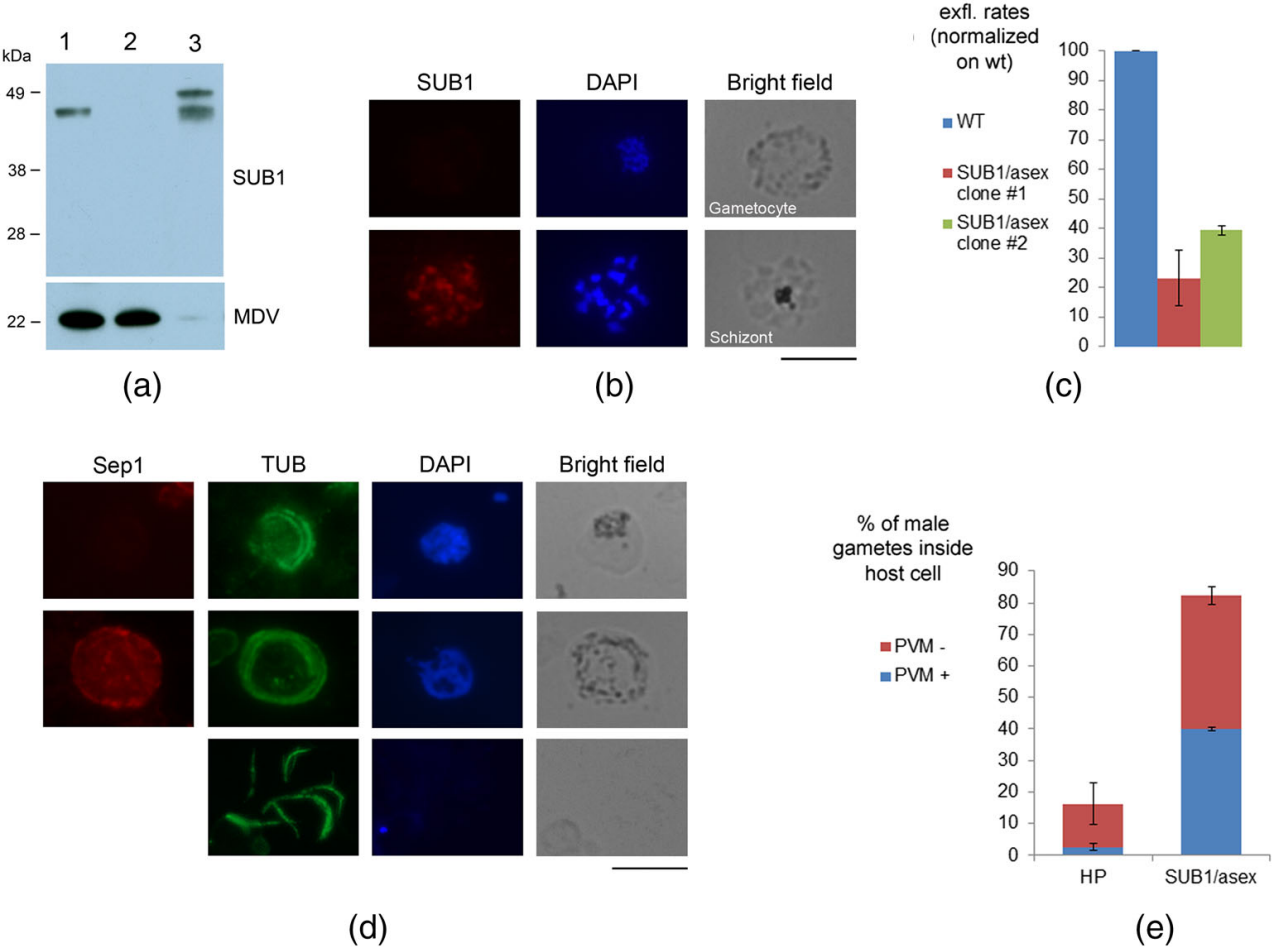
Stage‐specific knockdown of PbSUB1 expression results in a defect in male gamete egress. (a) Western blot analysis of the wt and the transgenic line SUB1/asex using anti‐SUB1. Anti‐MDV1 was used as a loading control of gametocyte samples. 1: purified wt gametocytes; 2: purified gametocytes from the SUB1/asex line; and 3: SUB1/asex mixed asexual stages. Interestingly, in wt gametocytes, only the final SUB1 maturation product was identified, in contrast to asexual stages, where both the processing products corresponding to Plasmodium falciparum p54 and p47 are detected (Suarez et al., 2013). (b) IFA of SUB1/asex line with polyclonal anti‐SUB1 antibody, showing a negative gametocyte and a positive schizont from the same in vitro culture. Scale bar 5 μm. (c) Exflagellation rates of two clones from the SUB1/asex line (at 20 min postinduction), expressed as a percentage of exflagellation rates in the wt line. Student's t test: P < .005 for Clone #2 and <.0002 for Clone #3. Three samples were analysed for each clone. (d) IFA of activated wt gametocytes at 20 min postinduction, with anti‐alpha‐tubulin, which stains male gamete flagella, and anti‐Sep1, a PVM marker. The image shows parasites from the wt line as an example of male gametes still inside the host erythrocyte with no PVM (top panel), male gametes still inside the host erythrocyte still owing an intact PVM (middle panel), and free male gametes (lower panel). Scale bar 5 μm. (e) Percentage of male gametes still inside the host cell, in the wt and SUB1/asex line Clone #2, at 20 min postinduction (Student's t test: P < .001). In blue, the amount of nonreleased male gametes still owing an intact PVM, in the wt and SUB1/asex line Clone #2 (Student's t test: P < .011)

To confirm PbSUB1 function in gametocytes, we generated a second transgenic *P*. *berghei* line, named SUB1/asex, in which PbSUB1 expression was selectively abolished in gametocytes. To do this, the endogenous *pbsub1* promoter was substituted with the promoter of the apical membrane antigen 1 (*ama1*; Figure S5a). The transgenic line was cloned and positive clones were identified by diagnostic PCR analysis (Figure S5b). AMA1 is expressed at approximately the same developmental stage as PbSUB1 in mature asexual stages but is not expressed in gametocytes (Kocken et al., 1998). The SUB1/asex line was characterised by IFA and western blot analysis. In both cases, PbSUB1 was found to be expressed in transgenic schizonts in a similar manner to wt parasites, whereas it was not detected in SUB1/asex gametocytes (Figure 4a,b). Also, in SUB1/asex exflagellation supernatants, SERA3 processing was inhibited, confirming the results previously obtained in the SUB1/prod line (Figure S6). To investigate the role of PbSUB1 in gametogenesis, two independent clones of the SUB1/asex line and wt parasites were induced to form gametes and the efficiency of male gamete formation was measured by counting exflagellation centres 20 min postactivation. As shown in Figure 4c, there was a significant reduction of male gamete exflagellation rates in the transgenic parasites compared with wt parasites. This experiment was conducted independently twice (two biological replicates), on each occasion performed in triplicate. To investigate whether the observed phenotype was maintained upon even longer induction times, an activation time course experiment was performed with parasites from the SUB1/asex clone 2 and wt parasites, quantifying exflagellation centres at 10, 15, 20, and 30 min postinduction. This experiment, conducted using three technical replicates, showed that the numbers of exflagellating males were still significantly lower than the wt control even at 30 min postinduction (Figure S6a).

We next investigated the ability of SUB1/asex gametocytes to release mature male gametes quantifying by IFA released and nonreleased male gametes 20 min after induction, using an antitubulin antibody to stain flagella. The presence or absence of the PVM in nonreleased male gametes was evaluated using an antibody against the PVM marker small exported protein 1 (Sep1; Figure 4d). These experiments, conducted in two biological replicates each performed in triplicate, showed that the proportion of nonreleased male gametes was significantly higher in the SUB1/asex line compared with wt parasites (Figure 4e). In addition, the proportion of nonreleased male gametes still possessing an intact PVM was significantly higher in the SUB1/asex line than in the wt parasites (Figure 4e). Taken together, these results indicate a defect in PVM rupture of SUB1/asex male gametes and are consistent with the previously described role of SUB1 in schizont PVM rupture (Thomas et al., 2018). Because the AMA1 promoter is active in the mosquito stages (Schmidt‐Christensen et al., 2008), phenotypic analysis of the SUB1/asex transgenic line could not be taken further.

## 3 DISCUSSION

In this study, we analysed the expression profile and function of the subtilisin‐like serine protease PbSUB1 in *P*. *berghei* sexual stages. We first examined *sub1* gene expression and showed that the *sub1* promoter is bipartite, with one region driving gene expression in the sexual stages, with a peak in activated male gametocytes, and another segment necessary to drive expression also in asexual stages. We then investigated the subcellular localisation of the protease in the sexual stages, showing that PbSUB1 is expressed specifically in male gametocytes and localises to specialised secretory organelles called MOBs, previously shown to be involved in the exflagellation process (Olivieri et al., 2015; Tachibana et al., 2018). We have also demonstrated here that the enzymatic activity of PbSUB1 is important for male gamete egress from the host cell. In *P*. *berghei*, three proteins localising to OBs have been previously reported to play a role in gamete emergence: *P*. *berghei* gametocyte 377 protein (PbG377; de Koning‐Ward et al., 2008; Olivieri et al., 2015), gamete egress and sporozoite traversal protein (PbGEST; Talman et al., 2011), and MDV1 (Ponzi et al., 2009). PbG377 is expressed only in female gametocytes and plays a role in OB biogenesis (Alano et al., 1995; Severini et al., 1999): its absence causes a dramatic reduction in OB size, as well as a slight delay in female gametocyte egress (de Koning‐Ward et al., 2008; Olivieri et al., 2015). PbGEST and MDV1 are expressed in both genders and localise to OBs/MOBs (Ponzi et al., 2009; Talman et al., 2011). Genetic deletion of PbGEST and MDV1 strongly affects gamete egress from the host cell. In these parasites, gametocytes of both genders fully develop inside RBCs but remain entrapped within the PVM after activation of gametogenesis. In parasites lacking PbSUB1 in gametocytes (SUB1/asex transgenic line), we observed that at 20 min postactivation, around 40% of SUB1/asex male gametes still had an intact PVM, compared with only 2% in the wt line. This is consistent both with the described function of SUB1 in asexual stages, being a protease essential for schizont PVM rupture (Thomas et al., 2018), and with the role of OB/MOBs in gamete egress. Interestingly, PbSUB1 is the only OB/MOB resident protein described to be expressed also in asexual stages, where it localises to exonemes, secretory vesicles involved in parasite egress (Yeoh et al., 2007). This suggests that MOBs and exonemes may belong to the same category of vesicles and possibly display some overlap in their function and protein content and share the same localisation signals. In conclusion, we described a novel specific marker of MOBs and showed that it plays a role in gamete egress.

We also showed here for the first time that the PbSUB1 substrate PbSERA3 is expressed in gametocytes and processed by PbSUB1 upon gametocyte activation. SERA3 shows a localisation compatible with the PV both in schizonts and male gametocytes, consistent with the localisation of its P. falciparum orthologue (Ruecker et al., 2012; Suarez et al., 2013; Tawk et al., 2013), and a punctate cytoplasmic pattern in female gametocytes. In nonactivated gametocytes, SERA3 is mostly present in the unprocessed form of 130 kDa and in the partial maturation product of 55 kDa, whereas in supernatants from activated gametocytes, the most abundant band is the final maturation product of 48 kDa, thus demonstrating that PbSUB1 only processes its substrate PbSERA3 when it is discharged into the PV.

SUB1 is already considered a promising drug target for malaria chemotherapy, being essential in both hepatic and asexual blood stages of the *Plasmodium* life cycle. Several P. falciparum SUB1‐specific inhibitors have been developed (Gemma et al., 2012; Giovani et al., 2014; Kher et al., 2014) and in some cases were shown to be effective in inhibiting parasite emergence from erythrocytes, indicating that SUB1 is a druggable target. In addition, the P. falciparum PfSUB1 and Plasmodium vivax PvSUB1 x‐ray crystal structures were recently made available (Giganti et al., 2014; Withers‐Martinez et al., 2014). Because no structurally similar proteases have been identified in the human genome, the development of highly selective SUB1 inhibitors appears feasible. Here, we have demonstrated that PbSUB1 plays an important role in disease transmission, making the protease an even more interesting therapeutic candidate, given the current focus on malarial molecular targets suitable for transmission‐blocking drug development. Exploring the possible presence of this protease in the sexual stages of P. falciparum and investigating its role in these stages would be therefore a very promising research topic.

## 4 EXPERIMENTAL PROCEDURES

### 4.1 DNA construct generation

To produce the SUB1/prom constructs, the putative *pbsub1* 3' UTR, spanning from −36 to +766 bp from the stop codon, was amplified by genomic DNA with the primer pair SUB1‐3'UTR_for and SUB1‐3'UTR_rev (Table S1), digested with NarI/SacI and cloned in the plasmid pBAT‐GFP (Kooij et al., 2012), kindly provided by Taco Kooij, digested with the same enzymes. Two putative *pbsub1* promoter regions, respectively named SUB1‐prom1 and SUB1‐prom2, respectively, of 830 bp (from −1,279 to −450 bp from ATG) and 1,956 bp (from −2,385 to −450 bp from ATG, partially overlapping the EST of gene located upstream of *sub1*) were amplified by genomic DNA. The amplification products were respectively digested with SpeI/NdeI and SacII/NotI and cloned into the pBAT‐GFP, containing the putative *pbsub1* 3'UTR, digested with the same enzymes. The obtained constructs were respectively named pSUB1‐prom1 and pSUB1‐prom2.

In order to produce the *Sub1/prod* construct, MDV1 N‐terminal 90 aa were amplified by genomic DNA with the primer pairs MDV1 and digested with EcoRI/NarI, and PbSUB1 prodomain was amplified with the primer pair SUB1‐prod and digested with NarI/BssHII. Both PCR fragments were simultaneously cloned into a plasmid derived from the plasmid pDEF‐SSU‐hDHFR alias pL0008, a plasmid containing sequences for integration in the ribosomal DNA (kindly provided by Prof. C.J. Janse) digested with EcoRI‐BssHII. The coding regions were sequenced with the primers prod_seq1 to prod_seq5 (Table S1).

MDV1 promoter region, spanning from −923 to −133 bp from the ATG, was then amplified with the primer pair MDV1‐pr, digested with EcoRI/PstI, and cloned into the resulting plasmid, also digested with the same enzymes.

To produce the *Sub1/asex* construct, a gene targeting construct was made by modifying the plasmid pL1313 designed for targeted gene disruption by double crossover homologous recombination (Laurentino et al., 2011). A 956‐bp 5′‐targeting region and a 1,006‐bp 3′ targeting region were amplified from *P*. *berghei* genomic DNA using primer pairs sub1‐swap‐L and sub1‐swap‐R (Table S1) and cloned into the pL1313 plasmid digested with KpnI‐HindIII and NcoI‐SacII, respectively. The 5′‐targeting region includes a DNA sequence upstream of *pbsub1* start codon (−1,420 bp to −477), whereas the 3′ targeting region includes part of the coding sequence (−107 to +881 bp from *pbsub1* start codon). The coding region was sequenced with the primers SUB1_seq1 and SUB1_seq2 (Table S1) to confirm that no mutations were introduced during the amplification process.

The AMA1 promoter, amplified by genomic DNA with the primer pairs sub1‐swap‐prAMA1, was then cloned into the resulting plasmid digested with EcoRV/BamHI. This promoter region had previously been used and characterised in P. falciparum (Olivieri et al., 2011).

### 4.2 Transgenic parasite production and cloning

*P*. *berghei* ANKA high gametocyte producer cloned line (8417HP) was maintained in Swiss mice. Synchronous infections were established in CD1 mice by intravenous injection of purified infective schizonts (Janse & Waters, 1995). Blood was collected by heart puncture under anaesthesia and leukocytes were removed using Plasmodipur leukocyte filters (Euro‐Diagnostica). Schizont‐ or gametocyte‐infected erythrocytes were separated from uninfected cells through 14.3% Nycodenz density gradient centrifugation (Janse & Waters, 1995).

Transfection and selection of transformed parasites was performed using standard genetic modification technologies for *P*. *berghei* (Janse et al., 2006) using *P*. *berghei* HP as the parent parasite line. Cloned parasite lines were obtained by limiting dilution.

Plasmid integration in the transgenic lines was confirmed by diagnostic PCR reactions with the primers prAMA1‐int‐for and SUB1_seq2 for the SUB1/asex line and diag‐mdv‐for diag‐mdv‐rev (Table S1).

### 4.3 In vivo evaluation of GFP fluorescent parasites

Parasites from the SUB1/prom1 and SUB1/prom2 lines were synchronised by intravenous injection of purified infective schizonts and analysed at the fluorescence microscope to investigate GFP expression profile. Nuclei were stained with Hoechst. GFP fluorescence was analysed in synchronous schizonts at different time points (20, 21, and 22 hr postinvasion) and blood smears were produced at each time point to confirm by IFA with anti‐PbSUB1 that the sample included PbSUB1‐expressing schizonts. In order to investigate GFP expression in the sexual stages, synchronous gametocytes at 29 hpi were collected and analysed at the fluorescence microscope. The transgenic lines SUB1/prom1 and SUB1/prom2 showed GFP fluorescence with similar intensity. Scale bar 5 μm.

### 4.4 In vivo evaluation of male gamete egress

Transgenic and wt parasites were induced to form gametes in RPMI pH 8.0 supplemented with 50‐μM xanthurenic acid and kept at 20°C. Male gamete formation was measured by counting exflagellation centres at the optical microscope. Blood collected from the mouse tail was diluted 1:25 and counted in Neubauer chamber in a total volume 0.1 μl roughly corresponding to 24,000 erythrocytes. Exflagellation centre counts were normalised on the number of male gametocytes per 24,000 erythrocytes in each sample analysed, quantified on Giemsa‐stained blood smears with an optical microscope.

### 4.5 IFA and western blot

For IFA, air‐dried thin blood films were fixed in 4% paraformaldehyde for 30 min, permeabilised for 10 min with 0.1% Triton X100, and then blocked for 1 hr with 3% (*w*/*v*) bovine serum albumin. Samples were then probed for 1 hr with the HA‐specific mAb 3F10 (Roche) diluted 1:200; and/or a rabbit polyclonal antiserum raised against PbSUB1 (Suarez et al., 2013) diluted 1:100; and/or a rabbit polyclonal antiserum raised against the PV marker Sep1 (Birago et al., 2003) diluted 1:100; and/or a mouse polyclonal antiserum raised against the gender marker SE translocation diluted 1:100; and/or a mouse polyclonal antiserum raised against the OB‐marker MDV1 diluted 1:500; and/or a monoclonal mouse anti‐Tubulin antibody (Sigma) diluted 1:400; and/or a mouse polyclonal antiserum raised against PbSERA3 diluted 1:50 (Suarez et al., 2013). Samples were then washed for 5 min in PBS before incubation with an FITC or Rhodamine‐conjugated antimouse and/or antirabbit IgG (Thermo Scientific) diluted 1:400. Nuclei were stained with 4′,6‐diamidino‐2‐phenylindole and samples were mounted in Vectashield (Vector Laboratories). Pearson's correlation coefficients were calculated using the Coloc2 plugin of Fiji (Schindelin et al., 2012).

The amount of nonreleased male gametes was calculated by dividing the amount of circular flagella surrounded by a visible membrane in bright field by the sum of circular flagella and free flagella divided by 8, the number of gametes produced by a single male gametocyte.

For western blot analysis, culture supernatants were centrifuged at 16,000 *g*, passed through 22‐μm filters, acetone precipitated, and suspended in SDS sample buffer, whereas parasite pellets were solubilised in SDS sample buffer. All samples were then subjected to SDS‐PAGE under reducing conditions, followed by transfer to nitrocellulose membrane (Sartorius). Antibodies used: rabbit polyclonal antiserum raised against PfSUB1 diluted 1:500, rabbit polyclonal antiserum raised against PbSub1 diluted 1:5,000, mouse polyclonal antiserum raised against PbSERA3 diluted 1:1,000 (Suarez et al., 2013), monoclonal mouse anti‐HA diluted 1:2,000 (Roche), mouse polyclonal antiserum raised against MDV1 diluted 1:2,000, and mouse polyclonal antiserum raised against14‐3‐3 diluted 1:1,000 (Lalle et al., 2011). The filters were then incubated with antimouse or antirabbit HRP‐conjugated antibodies (Pierce) and the immunocomplexes were visualised using chemioluminescence ECL detection system (Luminata Forte Western HRP Substrate, Millipore).

### 4.6 Ethics statement

The protocol for infecting mice with *P*. *berghei* was approved and carried out at the University of Perugia under the Italian Ministry of Health Licence 1192/2016‐PR awarded in 2016, under the guidelines D.lgs. n. 26/2014 that implements the directive 86/609/EEC from the European Union.

## Supporting information


**Fig. S1.**
**Generation of the transgenic lines SUB1/prom.** A. Schematic of SUB1/prom transgenic lines. Arrows indicate the primers used for diagnostic PCRs. Red: Sil6_for; blue: RT‐revGFP; green: Sil6_rev; yellow: pBAT‐DraIII‐bk. B. Diagnostic PCR for identification of clones of SUB1/prom transgenic lines. Primers used for specific amplification of the wt region: Sil6_for and Sil6_rev (primer couple a); expected size: 1313 bp. The 5′ integration event was confirmed with the primers Sil6_for and RT‐revGFP (couple b); expected sizes: prom1 = 2022 bp; prom2 = 1597 bp; prom3 = 2685 bp. The 3′ integration event was confirmed with the primers pBAT‐DraIII‐bk and Sil6_rev (couple c); expected size: 1138 bp. Lanes1–3: wt control, primer couples a, b and c respectively; lanes 4–6: SUB1/prom1, primer couples a, b and c respectively; M: molecular weight marker (Hyperladder 1 Kb, Bioline); lanes 7–9: SUB1/prom2, primer couples a, b and c respectively; lanes 10–12: SUB1/prom3, primer couples a, b and c respectively.

**Fig. S2.**
**Immunofluorescence assay confirming anti‐SUB1 antibody specificity.** The picture shows a gametocyte from a synchronous infection of the SUB1‐HA‐tagged line. Anti‐SUB1 fluorescence signal co‐localises with the anti‐HA‐tag one. Scale bar 5 μm.

**Fig. S3.**
**Schematic of the transgenic lines expressing an HA‐tagged extra copy of SUB1 prodomain in gametocytes and PCR proving the integration event.** A. Schematic of the transgenic line SUB1/prod. The SUB1/prod plasmid was integrated into the genomic 18S ribosomal RNA locus, previously successfully used to integrate constructs into the P. berghei genome (Gunderson et al., 1987; Janse et al., 2006). In red: −923 bp to −133 bp upstream of MDV1 ATG, used as a promoter region; green: sequence corresponding to the first 90 aminoacids from MDV1 N‐terminus, HA‐tag and SUB1 prodomain; yellow: 3'UTR from the set gene, previously successfully used to express reporter genes in P. berghei gametocytes (Pace et al., 2006). The size of the target sequence was chosen based on previous work in which a reporter gene was targeted to P. falciparum OBs by fusing it to 90 aa from the OB‐resident protein Pfg377 (Sannella et al., 2012). Coloured arrows indicate the primers used for diagnostic PCRs. Green: L739_for; red: L635‐like; blue: Set‐3'UTR_for; yellow: L740‐like. B. Diagnostic PCR for identification of clones of the SUB1/prod transgenic line. Primers used for specific amplification of the 5′ integration event: L739_for and L635‐like_rev (primer couple a), expected size: 2102 bp. Primers used to specifically amplify the 3′ integration event: Set‐3'UTR_for and L740‐like_rev (couple b), expected size: 2654 bp. Lanes1 and 2: wt control, primer couples a and b respectively; lanes 3 and 4: SUB1/prod clone #1, primer couples a and b respectively; M: molecular weight marker (Hyperladder 1 Kb, Bioline); lanes 5 and 6: SUB1/prod clone #2, primer couples a and b respectively.

**Fig. S4.**
**Characterisation of the HA‐tagged prodomain expression profile in the SUB1/prod transgenic line.** Left: A. Western blot analysis of gametocytes probed with anti‐HA‐tag antibody (A). Lane 1: parental wt line; lane 2: transgenic line SUB1/prod clone #1. Anti‐SUB1 was used as a loading control (panel B). The expected molecular weight of the MDV1‐ prodomain chimera is 35 kDa. Right: IFA of SUB1/prod line clone #1 with anti‐HA antibody, showing gametocytes and asexual parasites from in vitro culture and trophozoites and rings from tail blood. Anti‐SET antibody detects SET, which decorates parasite nuclei, is abundantly expressed in male gametocytes and is used as a gender marker. Scale bar 5 μm.

**Fig. S5.**
**Schematic representation of the SUB1/asex transgenic line and PCR proving the integration event.** A. Coordinates of exchange regions are indicated. Arrows indicate the primers used for diagnostic PCRs. Green: SUB1_‐821_for; red: SUB1_seq2; blue: sub1‐swap‐prAMA1_for. B. Diagnostic PCR for identification of clones of the SUB1/asex transgenic line. Primers used for specific amplification of the wt region: SUB1_‐821_for and SUB1_seq2 (primer couple a), expected size: 1,418 bp. Primers used to specifically amplify the integration event: sub1‐swap‐prAMA1_for and SUB1_seq2 (couple b), expected size: 1,900 bp. Lanes1 and 2: wt control, primer couples a and b respectively; lanes 3 and 4: parental mouse, primer couples a and b respectively; M: molecular weight marker (Hyperladder 1 Kb, Bioline); lanes 5 and 6: clone #1, primer couples a and b respectively; lanes 7 and 8: clone #2, primer couples a and b respectively; lanes 9 and 10: clone #3, primer couples a and b respectively.

**Fig. S6.**
**Exflagellation time course analysis and SERA3 processing in the SUB1/asex transgenic line.** A. Exflagellation rates at 10, 15, 20 and 30 minutes post induction in the wt and SUB1/asex lines (normalised on male gametocytes). Student's t‐test at 30 min: p < 0,005. Error bars represent +/− standard deviation of the mean value. B. Western blot analysis with anti‐PbSERA3. Extracts from 5 × 10^6^ wt gametocytes (1) and 5 × 10^6^ SUB1/asex gametocytes (2); wt exflagellation supernatants from 5 × 10^6^ gametocytes (3), and SUB1/asex exflagellation supernatants from 5 × 10^6^ gametocytes (4). Anti‐14‐3‐3 was used as a loading control.

Table S1.
PCR primer list.
