# Supplementary figures and images for: The Plasmodium berghei serine protease PbSUB1 plays an important role in male gamete egress
Source: Cell Microbiol. 2019 Apr 29;21(7):e13028. doi: 10.1111/cmi.13028 (PMC6766862; doi:10.1111/cmi.13028)

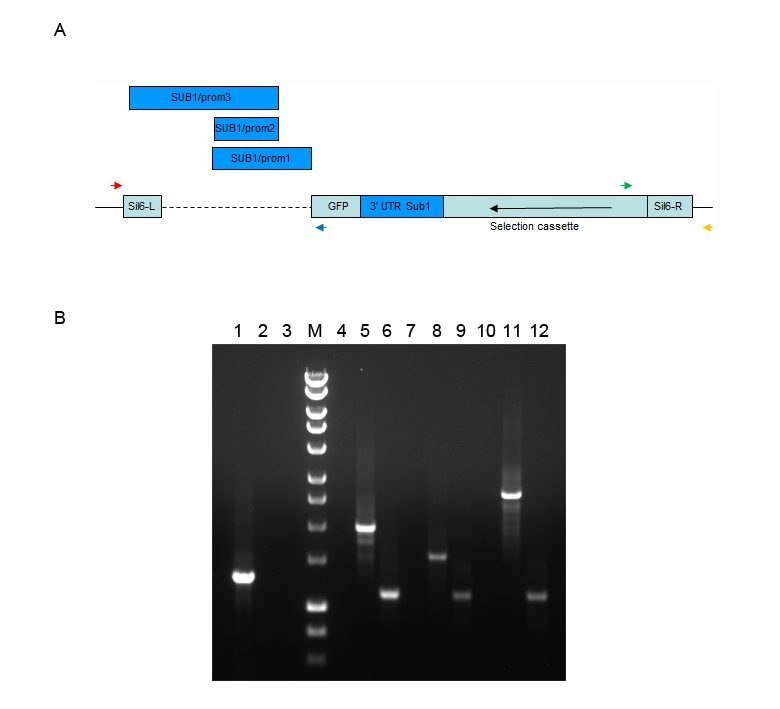

Supplement: Supplementary file 1 — Fig. S1. Generation of the transgenic lines SUB1/prom. A. Schematic of SUB1/prom transgenic lines. Arrows indicate the primers used for diagnostic PCRs. Red: Sil6_for; blue: RT‐revGFP; green: Sil6_rev; yellow: pBAT‐DraIII‐bk. B. Diagnostic PCR for identification of clones of SUB1/prom transgenic lines. Primers used for specific amplification of the wt region: Sil6_for and Sil6_rev (primer couple a); expected size: 1313 bp. The 5′ integration event was confirmed with the primers Sil6_for and RT‐revGFP (couple b); expected sizes: prom1 = 2022 bp; prom2 = 1597 bp; prom3 = 2685 bp. The 3′ integration event was confirmed with the primers pBAT‐DraIII‐bk and Sil6_rev (couple c); expected size: 1138 bp. Lanes1–3: wt control, primer couples a, b and c respectively; lanes 4–6: SUB1/prom1, primer couples a, b and c respectively; M: molecular weight marker (Hyperladder 1 Kb, Bioline); lanes 7–9: SUB1/prom2, primer couples a, b and c respectively; lanes 10–12: SUB1/prom3, primer couples a, b and c respectively. [file CMI-21-na-s001.tif]

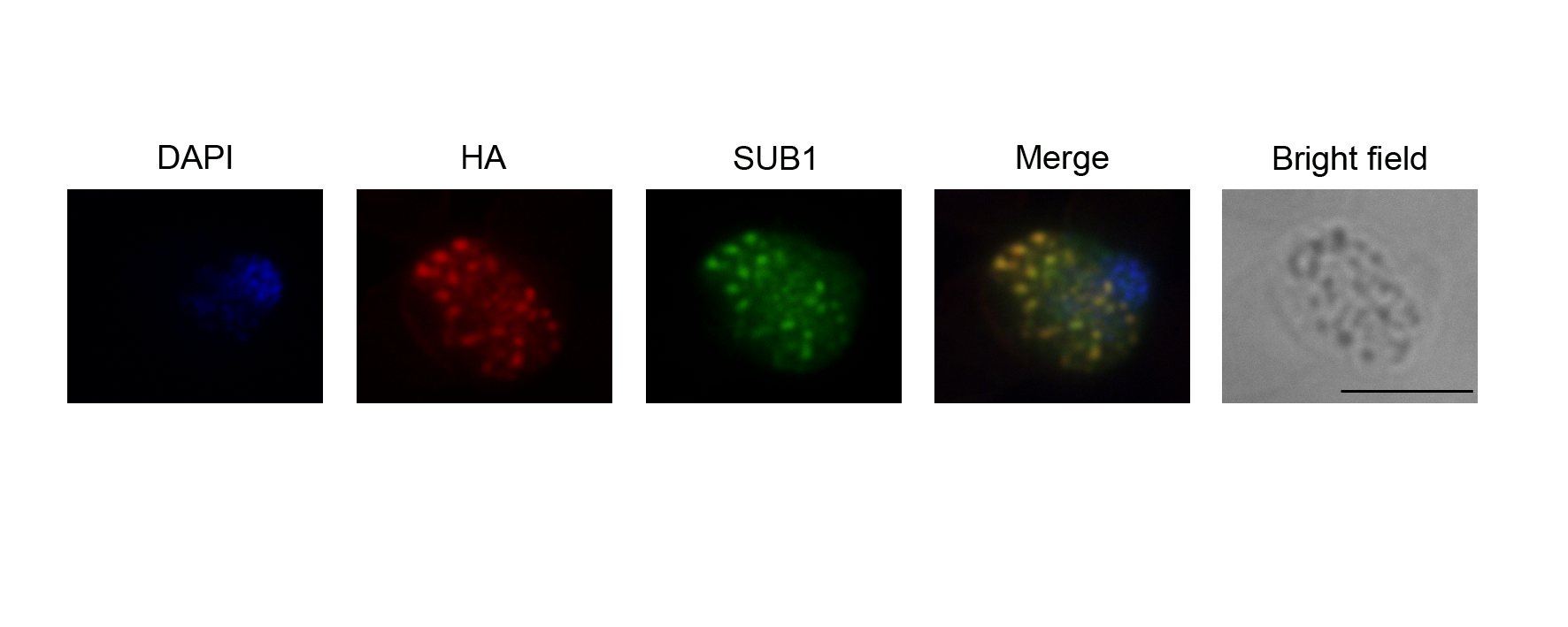

Supplement: Supplementary file 2 — Fig. S2. Immunofluorescence assay confirming anti‐SUB1 antibody specificity. The picture shows a gametocyte from a synchronous infection of the SUB1‐HA‐tagged line. Anti‐SUB1 fluorescence signal co‐localises with the anti‐HA‐tag one. Scale bar 5 μm. [file CMI-21-na-s002.tif]

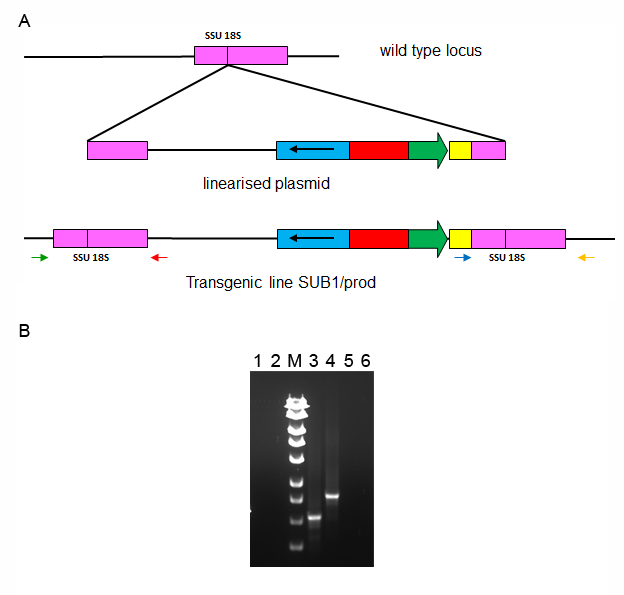

Supplement: Supplementary file 3 — Fig. S3. Schematic of the transgenic lines expressing an HA‐tagged extra copy of SUB1 prodomain in gametocytes and PCR proving the integration event. A. Schematic of the transgenic line SUB1/prod. The SUB1/prod plasmid was integrated into the genomic 18S ribosomal RNA locus, previously successfully used to integrate constructs into the P. berghei genome (Gunderson et al., 1987; Janse et al., 2006). In red: −923 bp to −133 bp upstream of MDV1 ATG, used as a promoter region; green: sequence corresponding to the first 90 aminoacids from MDV1 N‐terminus, HA‐tag and SUB1 prodomain; yellow: 3'UTR from the set gene, previously successfully used to express reporter genes in P. berghei gametocytes (Pace et al., 2006). The size of the target sequence was chosen based on previous work in which a reporter gene was targeted to P. falciparum OBs by fusing it to 90 aa from the OB‐resident protein Pfg377 (Sannella et al., 2012). Coloured arrows indicate the primers used for diagnostic PCRs. Green: L739_for; red: L635‐like; blue: Set‐3'UTR_for; yellow: L740‐like. B. Diagnostic PCR for identification of clones of the SUB1/prod transgenic line. Primers used for specific amplification of the 5′ integration event: L739_for and L635‐like_rev (primer couple a), expected size: 2102 bp. Primers used to specifically amplify the 3′ integration event: Set‐3'UTR_for and L740‐like_rev (couple b), expected size: 2654 bp. Lanes1 and 2: wt control, primer couples a and b respectively; lanes 3 and 4: SUB1/prod clone #1, primer couples a and b respectively; M: molecular weight marker (Hyperladder 1 Kb, Bioline); lanes 5 and 6: SUB1/prod clone #2, primer couples a and b respectively. [file CMI-21-na-s003.tif]

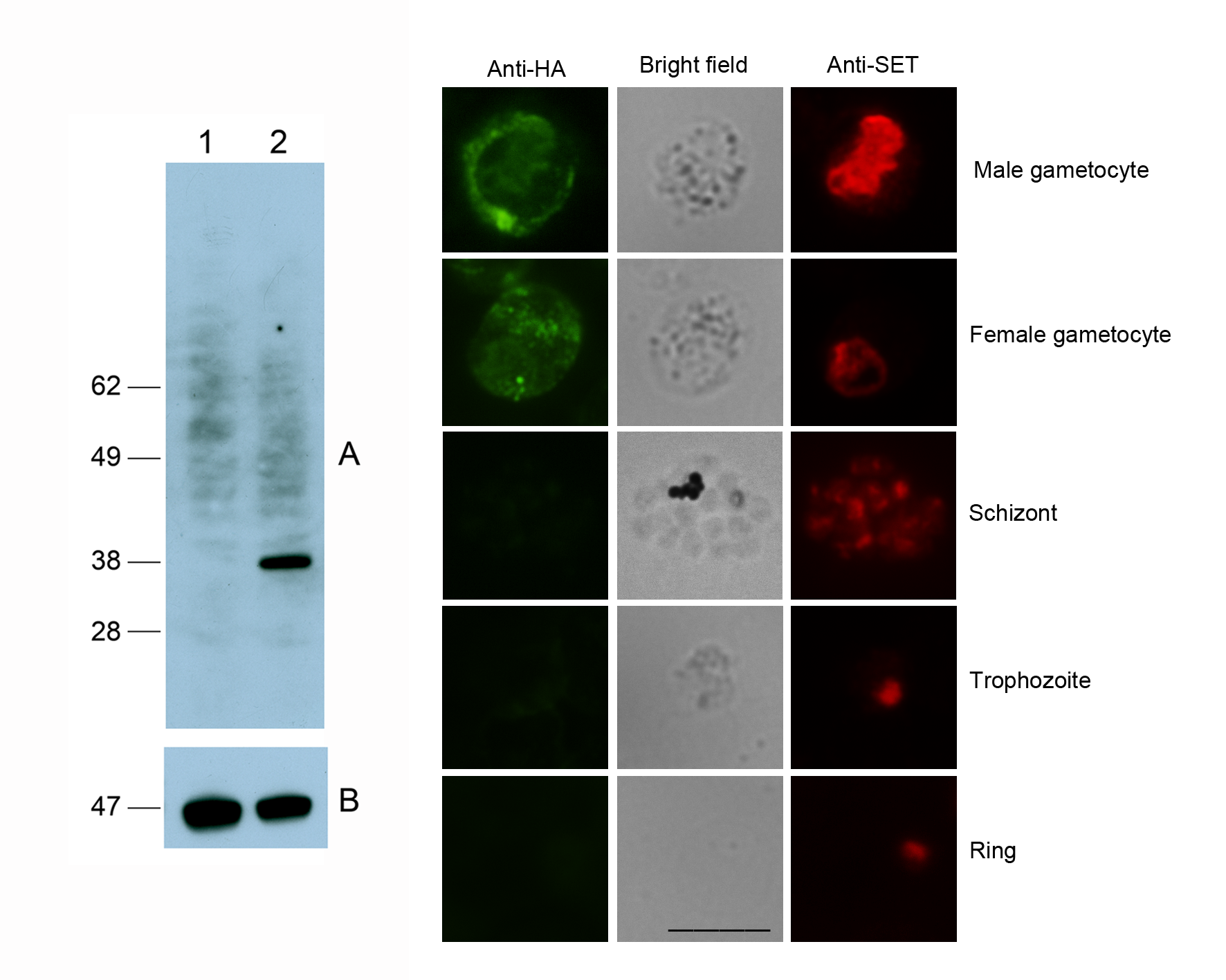

Supplement: Supplementary file 4 — Fig. S4. Characterisation of the HA‐tagged prodomain expression profile in the SUB1/prod transgenic line. Left: A. Western blot analysis of gametocytes probed with anti‐HA‐tag antibody (A). Lane 1: parental wt line; lane 2: transgenic line SUB1/prod clone #1. Anti‐SUB1 was used as a loading control (panel B). The expected molecular weight of the MDV1‐ prodomain chimera is 35 kDa. Right: IFA of SUB1/prod line clone #1 with anti‐HA antibody, showing gametocytes and asexual parasites from in vitro culture and trophozoites and rings from tail blood. Anti‐SET antibody detects SET, which decorates parasite nuclei, is abundantly expressed in male gametocytes and is used as a gender marker. Scale bar 5 μm. [file CMI-21-na-s004.tif]

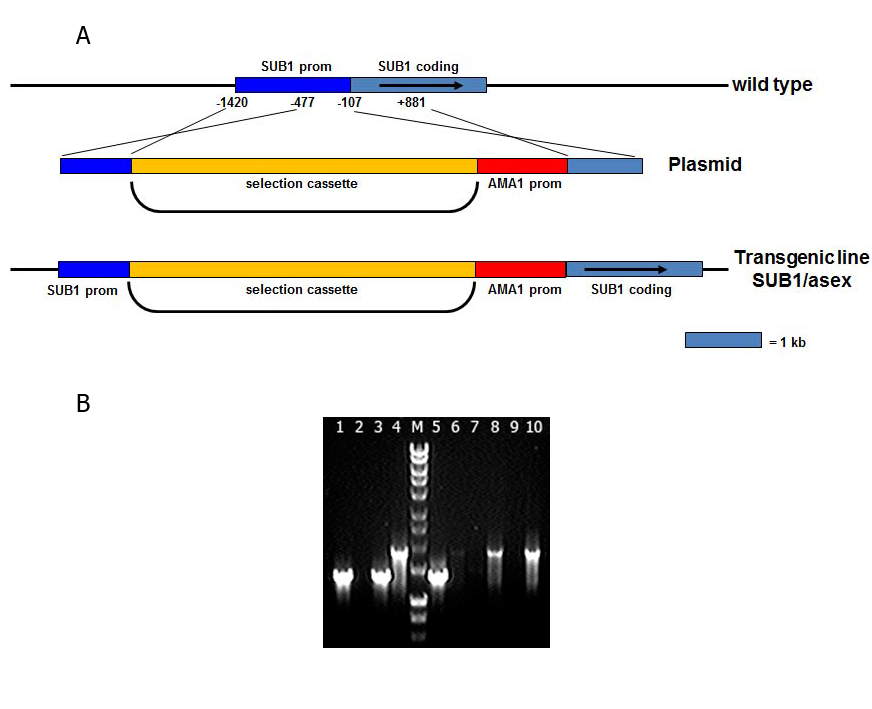

Supplement: Supplementary file 5 — Fig. S5. Schematic representation of the SUB1/asex transgenic line and PCR proving the integration event. A. Coordinates of exchange regions are indicated. Arrows indicate the primers used for diagnostic PCRs. Green: SUB1_‐821_for; red: SUB1_seq2; blue: sub1‐swap‐prAMA1_for. B. Diagnostic PCR for identification of clones of the SUB1/asex transgenic line. Primers used for specific amplification of the wt region: SUB1_‐821_for and SUB1_seq2 (primer couple a), expected size: 1,418 bp. Primers used to specifically amplify the integration event: sub1‐swap‐prAMA1_for and SUB1_seq2 (couple b), expected size: 1,900 bp. Lanes1 and 2: wt control, primer couples a and b respectively; lanes 3 and 4: parental mouse, primer couples a and b respectively; M: molecular weight marker (Hyperladder 1 Kb, Bioline); lanes 5 and 6: clone #1, primer couples a and b respectively; lanes 7 and 8: clone #2, primer couples a and b respectively; lanes 9 and 10: clone #3, primer couples a and b respectively. [file CMI-21-na-s005.tif]

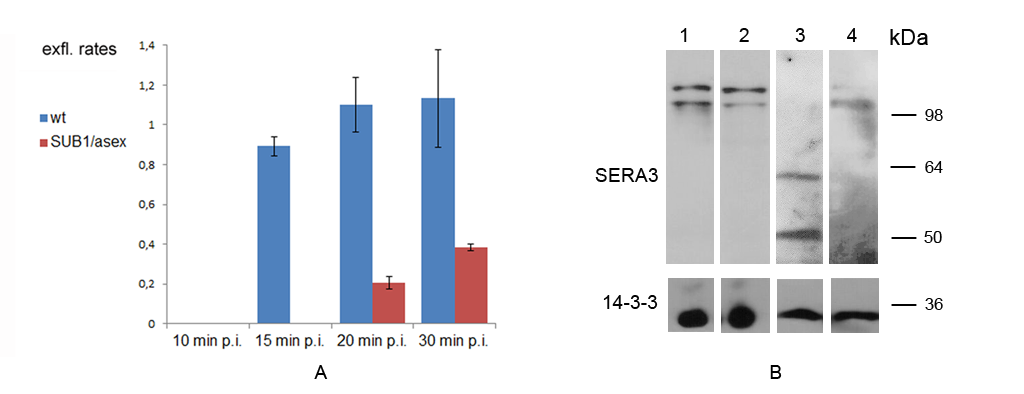

Supplement: Supplementary file 6 — Fig. S6. Exflagellation time course analysis and SERA3 processing in the SUB1/asex transgenic line. A. Exflagellation rates at 10, 15, 20 and 30 minutes post induction in the wt and SUB1/asex lines (normalised on male gametocytes). Student's t‐test at 30 min: p < 0,005. Error bars represent +/− standard deviation of the mean value. B. Western blot analysis with anti‐PbSERA3. Extracts from 5 × 10^6^ wt gametocytes (1) and 5 × 10^6^ SUB1/asex gametocytes (2); wt exflagellation supernatants from 5 × 10^6^ gametocytes (3), and SUB1/asex exflagellation supernatants from 5 × 10^6^ gametocytes (4). Anti‐14‐3‐3 was used as a loading control. [file CMI-21-na-s006.tif]
