# Supplementary material for: The Plasmodium berghei serine protease PbSUB1 plays an important role in male gamete egress
Source: Cell Microbiol. 2019 Apr 29;21(7):e13028. doi: 10.1111/cmi.13028 (PMC6766862; doi:10.1111/cmi.13028)
Supplement: Supplementary file 7 — Table S1. PCR primer list. [file CMI-21-na-s007.docx]

**Table S1. PCR primer list.**

| Primer name | Sequence |
| --- | --- |
| sub1-swap-L_for | gaccggtaccGAATCTCCCTATTTTGAGAATT |
| sub1-swap-L_rev | gaccaagcttCTCATAATTAACAACTGCTTACAA |
| Sub1-swap-R_for | gaccggatccccatggCCCCAAATTTGCCTGTATTGGTC |
| Sub1-swap-R_rev | gaccccgcggGGATGATTATAATCTATACCAC |
| sub1-swap-prAMA1_for | gaccgatatcgaattcgagtgtacaatttgcatagtgag |
| sub1-swap-prAMA1_rev | gaccggatccccatacaatacttatatatacacac |
| MDV1_for | gaccgaattccctaggATGAAGTGTATTAATATTCC |
| MDV1_rev | gaccggcgcctcgcgaagcataatcaggtacatcataaggataTGTAGCTAATTCAGAAGCTAG |
| SUB1-prod_for | gaccggcgccgacgtcgattataaggatgatgatgataagAATGATTTAATGAGTAAAGAAAAAG |
| SUB1-prod_rev | GaccgcgcgcaagcttTCAATCAGCTCCTACTAATTCATC |
| MDV1-pr_for | gaccctgcagctcgagCAACATTAAGTGATAATCGTAAC |
| MDV1-pr_rev | gaccgaattccgatagccctaatgaaaataattgcc |
| SUB1_seq1 | GTAACATGACAATTGATGATG |
| SUB1_seq2 | CAGCTCCTACTAATTCATCAG |
| prod_seq1 | gcttcgcgaggcgccgacg |
| prod_seq2 | cgtcggcgcctcgcgaagc |
| prod_seq3 | GTGAAAATCATGCAACTAG |
| prod_seq4 | CTGGATTATATGCATCTGC |
| prod_seq5 | GATGGAAGGCAATATACT |
| prAMA1-int-for | cgtacatctacgcattgt |
| diag-mdv-for | CAGCTCAATGCACATCTAGT |
| diag-mdv-rev | GCTAATTCAGAAGCTAGATC |
| SUB1-3’UTR_for | gaccggcgcctaaggatccCAAGGAATTCGCATGTGG |
| SUB1-3’UTR_rev | gaccgagctcaccggtCGAGCATGCAAACACACAC |
| SUB1_-1279_for | gaccactagtGGTCACTCTTGATGGGAA |
| SUB1_-821_for | gaccactagtCCAATGTTCCAAGTGTTTCG |
| SUB1_-450_rev | gacccatatggcggccgcGTCTTTATTATTTGTAGAG |
| SUB1_-25_rev | gacccatatggcggccgcGTACGAGAAATAGCTGTTTG |
| SUB1_-2385_for | gaccccgcggtctagaagatctGCTATTTGCGTACATCACAT |
| Sil6_for | GACAGCGCATATGATGGATGC |
| RT-revGFP | TTGTGCCCATTAACATCACC |
| Sil6_rev | CGAATACGCAATTTCTCAAAC |
| pBAT-DraIII-bk | CGTCCACCCCGTGTGAATATGC |
| 18S_RTPCR_for | TTTATTGGGAGATTGGTTTTGACGTTTATGTG |
| 18S_RTPCR_rev | AAGCATTAAATAAAGCGAATACATCCTTAC |
| SUB1_RTPCR_for | TATTGTCAAGTTTCAGCACCAG |
| SUB1_RTPCR_rev | AAGCATTAAATAAAGCGAATACATCCTTAC |
| L739_for | tttggatattttcatatat |
| L635-like_rev | cgaattggagctccactacg |
| Set-3’UTR_for | AAGCTTGCGCGCGATGATTA |
| L740-like_rev | ccacacaatctataattctg |
